# Entomological survey of sibling species in the *Anopheles funestus* group in Tanzania confirms the role of *Anopheles parensis* as a secondary malaria vector

**DOI:** 10.1186/s13071-024-06348-9

**Published:** 2024-06-17

**Authors:** Salum Abdallah Mapua, Badara Samb, Ismail Hassan Nambunga, Gustav Mkandawile, Hamis Bwanaly, Emmanuel Wilson Kaindoa, Joel Ouma Odero, John Paliga Masalu, Najat Feruz Kahamba, Emmanuel Elirehema Hape, Nicodem James Govella, Fredros Oketch Okumu, Frederic Tripet

**Affiliations:** 1 Environmental Health and Ecological Sciences Department, Ifakara Health Institute, P. O. Box 53, Morogoro, Tanzania; 2 Centre for Applied Entomology and Parasitology, School of Life Sciences, Keele University, Huxley Building, Keele, Staffordshire ST5 5BG UK; 3 Laboratoire d’Écologie Vectorielle et Parasitaire, Département de Biologie Animale, Faculté des Sciences et Techniques, Université Cheikh Anta Diop de Dakar, 5005 Dakar-Fann, BP Senegal; 4 School of Biodiversity, One Health and Veterinary Medicine, University of Glasgow, Glasgow, G61 1QH UK; 5 School of Pathology, Faculty of Health Sciences, University of the Witwatersrand, Johannesburg, South Africa; 6 School of Life Science and Bioengineering, The Nelson Mandela African Institution of Science and Technology, P. O. Box 447, Arusha, Tanzania; 7 Swiss Tropical and Public Health Institute, Kreuzgasse 2, 4123 Allschwil, Switzerland; 8 University of Basel, Basel, Switzerland

**Keywords:** Malaria, *Anopheles funestus*, *Plasmodium*, Tanzania

## Abstract

**Background:**

Malaria transmission in Tanzania is driven by mosquitoes of the *Anopheles gambiae* complex and *Anopheles funestus* group. The latter includes *An*. *funestus* s.s., an anthropophilic vector, which is now strongly resistant to public health insecticides, and several sibling species, which remain largely understudied despite their potential as secondary vectors. This paper provides the initial results of a cross-country study of the species composition, distribution and malaria transmission potential of members of the *Anopheles funestus* group in Tanzania.

**Methods:**

Mosquitoes were collected inside homes in 12 regions across Tanzania between 2018 and 2022 using Centres for Disease Control and Prevention (CDC) light traps and Prokopack aspirators. Polymerase chain reaction (PCR) assays targeting the noncoding internal transcribed spacer 2 (ITS2) and 18S ribosomal DNA (18S rDNA) were used to identify sibling species in the *An*. *funestus* group and presence of *Plasmodium* infections, respectively. Where DNA fragments failed to amplify during PCR, we sequenced the ITS2 region to identify any polymorphisms.

**Results:**

The following sibling species of the *An*. *funestus* group were found across Tanzania: *An*. *funestus* s.s. (50.3%), *An*. *parensis* (11.4%), *An*. *rivulorum* (1.1%), *An*. *leesoni* (0.3%). Sequencing of the ITS2 region in the nonamplified samples showed that polymorphisms at the priming sites of standard species-specific primers obstructed PCR amplification, although the ITS2 sequences closely matched those of *An*. *funestus* s.s., barring these polymorphisms. Of the 914 samples tested for *Plasmodium* infections, 11 *An*. *funestus* s.s. (1.2%), and 2 *An*. *parensis* (0.2%) individuals were confirmed positive for *P*. *falciparum*. The highest malaria transmission intensities [entomological inoculation rate (EIR)] contributed by the *Funestus* group were in the north-western region [108.3 infectious bites/person/year (ib/p/y)] and the south-eastern region (72.2 ib/p/y).

**Conclusions:**

Whereas *An*. *funestus* s.s. is the dominant malaria vector in the *Funestus* group in Tanzania, this survey confirms the occurrence of *Plasmodium*-infected *An*. *parensis*, an observation previously made in at least two other occasions in the country. The findings indicate the need to better understand the ecology and vectorial capacity of this and other secondary malaria vectors in the region to improve malaria control.

**Graphical Abstract:**

**Figure:**
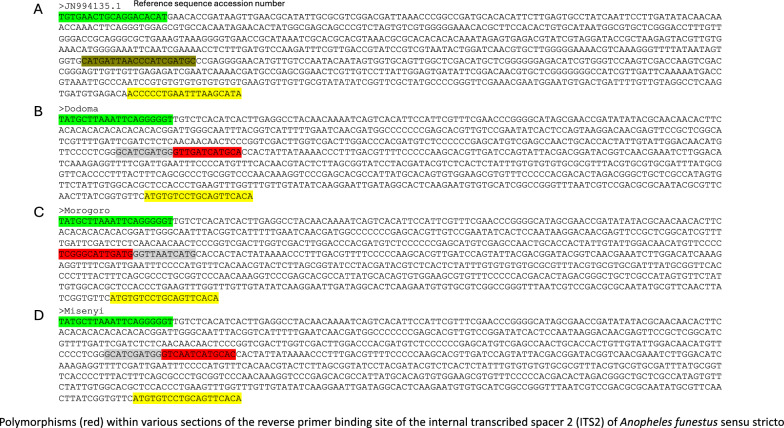

**Supplementary Information:**

The online version contains supplementary material available at 10.1186/s13071-024-06348-9.

## Background

Africa has witnessed significant progress in the fight against malaria from 2000, thanks to extensive vector control using insecticide-treated nets and indoor spraying, alongside improved diagnosis and treatment [1]. However, this progress began to stagnate around 2015, and currently, malaria causes ≤ 249 million cases and 608,000 deaths annually, predominantly in sub-Saharan Africa [2].

Other than the weak health systems and socio-economic conditions [3, 4], the persistent malaria burden in the region is exacerbated by several biological challenges, notably insecticide resistance [5, 6], anti-malarial drug resistance [7–9], failing diagnostics [10–13] and an invasive vector species, *Anopheles stephensi* [14–16]. The problem is compounded by human behaviour and lifestyles leading to inadequate protection during peak transmission periods, and insufficient community and stakeholder engagement in malaria prevention efforts [17–20]. Evidence also suggests that the traditional methods used to disrupt malaria transmission, notably insecticide treated bed nets (ITNs) and indoor residual spraying (IRS) are insufficient against certain vector species with atypical behaviours, such as those that do not bite or rest primarily indoors [21–23].

In east and southern Africa, the major malaria vectors are *Anopheles gambiae* sensu stricto, *Anopheles arabiensis* and *Anopheles funestus* mosquitoes [24, 25]. In most settings, *An*. *gambiae* s.s. and *An*. *funestus* mosquitoes have been historically dominating the malaria transmission [26–29]. However, recently, in some localities, such as in parts of western Kenya and south-eastern Tanzania, the wide coverage of ITNs likely in concert with environmental changes, appears to have significantly suppressed *An*. *gambiae* s.s. leaving *An*. *arabiensis* and *An*. *funestus* s.s. as the main drivers of transmission [30–33]. In Tanzania, *An*. *funestus* is now the dominant malaria vector across the country [34]. More detailed studies have revealed that even when outnumbered by *An*. *arabiensis*, *An*. *funestus* s.s. mediates over 90% of the ongoing malaria transmission in south-eastern Tanzania [31]. With *An*. *funestus* being highly anthropophilic, and in some settings having stronger resistance to public health insecticides compared with the other major malaria vectors [35], this vector species poses a significant challenge to the existing vector control interventions. It is noteworthy, that most studies have so far focused on only one member of the *An*. *funestus* group, i.e. *An*. *funestus* s.s. despite this species being one member of a large species complex [36].

The *An*. *funestus* group is thought to comprise 13 sibling species: *An*. *funestus* s.s.[25], *An*. *funestus*-like, *An*. *vaneedeni* [37], *An*. *parensis* [38–40], *An*. *rivulorum*[41], *An*. *rivulorum*-like [42], *An*. *leesoni*, *An*. *aruni*, *An*. *confusus*, *An*. *brucei*, *An*. *fuscivenosus* and *An*. *longipalpis* types A and C [43]. Of these, *An*. *funestus* s.s. is the most competent malaria vector in the group, though other sibling species, such as *An*. *rivulorum*, *An*. *leesoni* and *An*. *parensis* have also been reported to carry *Plasmodium falciparum* to lesser extent [37–39, 41, 43]. Despite these important observations, the species composition, distribution, and role in malaria transmission of the *An*. *funestus* group remains understudied, and several members of this group are likely to be misidentified. For instance, Ogola et al. [44] reported an unidentified sibling species within the *An*. *funestus* group in Kenya, and existing polymerase chain reaction (PCR) assays commonly return unamplified samples, including those morphologically confirmed as belonging to the group [45, 46]. More importantly, our understanding of the ecological dynamics and potential roles of these in perpetuating persistent malaria transmission remains limited.

To bridge these knowledge gaps, our research team initiated and implemented a cross-country survey of malaria vectors aimed at determining the species composition, spatial distribution, and the relative contribution of different *Anopheles* spp. to malaria transmission in mainland Tanzania. This paper presents the results from the initial phase of these surveys, covering 14 districts in 12 regions across Tanzania.

## Methods

### Study area

**Fig. 1:**
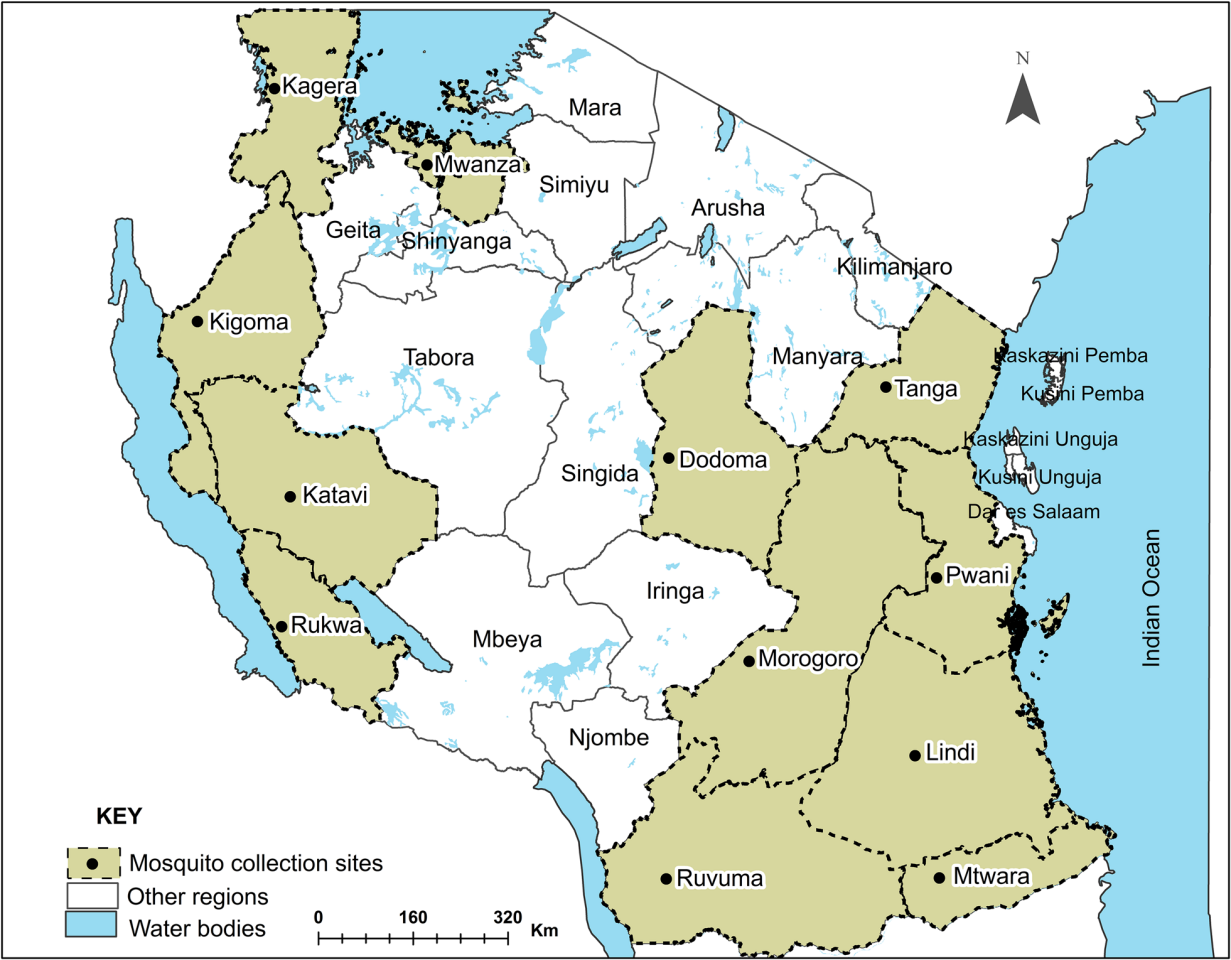
Map of Tanzania showing the regions where *Anopheles funestus* mosquitoes were collected

Mosquitoes were collected from 14 districts in 12 administrative regions across Tanzania mainland (Fig. 1). Tanzania has a broadly tropical climate, with four primary climatic zones: the hot and humid coastal plain (i.e. Pwani, Tanga, Lindi and Mtwara), the semi-arid central plateau (i.e. Dodoma, Kigoma, Katavi and Rukwa), the high rainfall lake regions (i.e. Kagera and Mwanza) and the cooler highlands (i.e. Morogoro and Ruvuma). On the Tanzanian coast and offshore islands, temperatures typically fluctuate between 27 °C and 29 °C. In the central, northern, and western regions, temperatures vary between 20 °C and 30 °C. The extended rainy season spans from March to May, while the shorter rainy season extends from October to early December, with the dry season lasting from June to September. Overall, annual rainfall ranges from 550 mm in the central areas to 3690 mm in certain parts of the southwestern highlands [47]. In most of these districts, the majority of the rural households are subsistence farmers [48, 49]. Malaria prevalence in children under the age of 5 years differs significantly in the study area, with the highest in the north-western (i.e. Kagera and Kigoma) and south-eastern regions (i.e. Mtwara and Lindi) to less than 1% in the central region (i.e. Dodoma)[50].

The data collection sites are shown in Fig. 1. Specific districts were: Misenyi in Kagera, Kakonko and Kibondo in Kigoma, Chamwino in Dodoma, Ulanga and Kilombero in Morogoro, Tunduru in Ruvuma, Bagamoyo in Pwani, Nkasi in Rukwa, Tanganyika in Katavi, Misungwi in Mwanza, Mtama in Lindi, Mahurunga in Mtwara and Muheza in Tanga. These collection sites represent diverse geographical regions, including the hot and humid coastal plain (i.e. Bagamoyo, Muheza, Mtama and Mahurunga), the semi-arid central plateau (i.e. Chamwino, Kakonko, Kibondo, Tanganyika and Nkasi), the high rainfall lake regions (i.e. Misenyi and Misungwi) and the cooler highlands (i.e. Kilombero, Ulanga and Tunduru).

### Mosquito collection and processing

Mosquito collections, conducted as part of a larger project on the population genetics of *An*. *funestus* sensu lato, were sporadic and completed between December 2018 and December 2022. These collections spanned both dry and wet seasons. Whereas multiple mosquito species were collected, only *An*. *funestus* s.l. are used in this analysis. In each of the districts, at least two houses were selected upon consent from the household heads and used for the collection of adult mosquitoes. Centres for Disease Control and Prevention (CDC) light traps [51] and Prokopack aspirators [52] were used to sample indoor host-seeking and resting mosquitoes, respectively. The overall sampling approach had been specifically designed to collect *An*. *funestus* s.l for population genetics studies, and was specifically focused on indoor collections, with no outdoor trapping in this phase of the study. Thus, mosquitoes were morphologically sorted to the species level and females of *An*. *funestus* s.l individually packed in an Eppendorf tube with 80% ethanol. In addition, in some locations, such as Dodoma, Tanga and Morogoro regions, where sampling of adult *An*. *funestus* s.l was insufficient, larval collections were conducted using standard larval dippers [53]. The collected larvae were reared to adults as previously described [54], then also sorted by taxa as above.

### Extraction of genomic DNA

Genomic DNA was extracted from the heads and thoraxes of collected mosquitoes using DNAzol method [55]. Bead Ruptor 96 well plate homogenizer (OMNI international, Kennesaw, GA, USA) was used for homogenization and the resultant DNA pellets were eluted in 50 µl of Tris–ethylenediaminetetraacetic acid (EDTA) buffer.

### Identification of the sibling species in the *An*. *funestus* group and detection of *Plasmodium* spp. infections

A cocktail of species-specific primers for the identification of the sibling species in the *An*. *funestus* group was used, as previously described by Koekemoer et al. [56]; with a slight adaptation to include a primer for *An*. *rivulorum*-like (Table 1) in the cocktail [42]. A nested PCR assay was used for the detection of the *Plasmodium* spp., of which the first round of the PCR included universal forward and reverse primers for 18S rDNA *Plasmodium* spp. (Table 2) regardless of species; followed by a second round using the amplicon from the first round as DNA template. Species-specific primers for *Plasmodium falciparum*, *Plasmodium ovale*, *Plasmodium vivax* and *Plasmodium malariae* were used in the second round (Table 2).

**Table 1:** Primers for PCR detection of *Anopheles funestus* group sibling species

Primer orientation	Primer sequence	Sibling species	Size of the PCR-product (bp)
Universal forward	TGTGAACTGCAGGACACAT	–	–
FUN reverse	GCATCGATGGGTTAATCATG	*An*. *funestus* s.s	505
VAN reverse	TGTCGACTTGGTAGCCGAAC	*An*. *vaneedeni*	587
RIV reverse	CAAGCCGTTCGACCCTGATT	*An*. *rivulorum*	411
PAR reverse	TGCGGTCCCAAGCTAGGTTC	*An*. *parensis*	252
LEES reverse	TACACGGGCGCCATGTAGTT	*An*. *leesoni*	146
RIVLIKE reverse	CCGCCTCCCGTGGAGTGGGGG	*An*. *rivulorum*-like	313

**Table 2:** Primers for a nested PCR detection of *Plasmodium* infection and species

PCR detection	Primer orientation	Primer sequence	Size of the PCR-product (bp)
*Plasmodium* infection detection	Forward	AGTGTGTATCAATCGAGTTTC	783–821
	Reverse	GACGGTATCTGATCGTCTTC	783–821
*Plasmodium* species-specific detection	Forward (universal)	CTATCAGCTTTTGATGTTAG	–
	*P*. *falciparum* reverse	GTTCCCCTAGAATAGTTACA	344
	*P*. *vivax* reverse	AAGGACTTCCAAGCC	457
	*P*. *ovale* reverse	CCAATTACAAAACCATG	202
	*P*. *malariae* reverse	TCCAATTGCCTTCTG	241

### Further analysis of the internal transcribed spacer 2 (ITS2) region in non-amplified *An*. *funestus* samples to investigate polymorphisms

A total of ten samples underwent cloning and sequencing, employing the following primers: ITS2A: 5′ TGT GAA CTG CAG GAC ACA T 3′ (forward) and ITS2B: 5′ TAT GCT TAA ATT CAG GGG GT 3′ (reverse). The PCR reaction mixture, conditions and procedures for the thermal cycling and electrophoresis were similar to those described earlier. The amplicons (approximately 840 base pairs) were excised from the gel and cleaned using Wizard^®^ SV Gel and PCR Clean-Up System (Catalogue number: #A9281, Promega). The purified product was cloned using a plasmid vector pJET1.2/blunt (CloneJET PCR Cloning Kit, Catalogue number: #K1231, Thermo Scientific). The resulting recombinant plasmid DNA was isolated and purified (QIAprep Spin Miniprep Kit, Catalogue number #27106, Qiagen) and sent for sequencing. Sequencing of the recombinant plasmid DNA was carried out using the reverse PJET1.2 primer (5′-AAGAACATCGATTTTCCATGGCAG-3′). Plasmid primer regions trimming, sequence alignment and analysis were performed using SeaView software [57].

### Data analysis

The data collected from the field included the number of traps used, the number of collection days, and the mosquitoes captured per trap, facilitating the calculation of trap nights (defined as the product of the number of traps and collection days). The annual entomological inoculation rate (EIR) was determined by multiplying human biting rates and *Plasmodium* sporozoite prevalence, then adjusted for 365 days [31]. A coefficient of 0.68 was used for conversion of the sampling efficiency of the CDC light trap relative to human landing catch (HLC) [31, 46]. Mosquitoes collected using Prokopack aspirators and from larval collections were excluded from the EIR calculation, as these methods do not accurately reflect host-seeking behaviour or the potential for infectivity. Basic Local Alignment Search Tool for nucleotides (BLASTn) analysis [58] was used to identify and characterize nucleotide sequences by finding homologous sequences in the National Center for Biotechnology Information nucleotide (NCBI nt) database. The top hits were retrieved and analysed to determine sequence similarity, alignment scores and query coverage. For the alignment and analysis of ITS2 sequences cloned from PCR-negative samples, SeaView software [57] was utilized.

## Results

### Species composition and distribution

A total of 1092 *An*. *funestus* s.l were analysed, of which 549 (50.3%) were *An*. *funestus* s.s., 124 were *An*. *parensis* (11.4%) and 12 were *An*. *rivulorum* (1.1%; Table 3). No *An*. *vaneedeni* or *An*. *rivulorum*-like were found during this study. While the other species, *An*. *funestus* s.s. and *An*. *rivulorum* were more widespread in the study sites, the *An*. *parensis* samples were found most abundantly in the central and northern regions (Table 3; Dodoma and Mwanza). There were 404 samples (37%) for which the DNA fragments did not amplify during the PCR (Table 3). Subsequent cloning and sequencing of the ITS2 region in these non-amplified samples revealed multiple polymorphisms within the reverse primer’s priming region specific to *An*. *funestus* s.s. (examples are shown in Additional file 1). The ITS2 sequences were found to be similar to those of *An*. *funestus* s.s., with the exception of polymorphisms within the priming site. BLASTn analysis revealed that the highest identity was 99.5%, considering sequences with 100% query coverage. The analysis confirmed that these sequences closely matched *An*. *funestus* s.s. sequences from the NCBI nt database.

**Table 3:** Composition and distribution of *Anopheles funestus* mosquitoes in mainland Tanzania

*An*. *funestus* group									
Region	District	No. tested	*An*. *funestus* s.s	*An*. *vaneedeni*	*An*. *parensis*	*An*. *rivulorum*	*An*. *rivulorum*-like	*An*. *leesoni*	Non-amplified
Dodoma	Chamwino	50	0	0	27	0	0	1	22
Kigoma	Kibondo	41	0	0	0	0	0	0	41
Kigoma	Kakonko	32	29	0	0	0	0	0	3
Tanga	Muheza	80	0	0	1	0	0	0	79
Kagera	Misenyi	48	42	0	0	0	0	0	6
Ruvuma	Tunduru	49	49	0	0	0	0	0	0
Morogoro	Ulanga (Kilisa)	21	0	0	0	0	0	1	20
Pwani	Bagamoyo	78	42	0	0	9	0	0	27
Mwanza	Misungwi	100	0	0	96	0	0	0	4
Katavi	Tanganyika	100	21	0	0	0	0	0	79
Rukwa	Nkasi	100	100	0	0	0	0	0	0
Lindi	Mtama	100	99	0	0	1	0	0	0
Mtwara	Mahurunga	100	100	0	0	0	0	0	0
Total_a_		1092	549 (50.3%)	0	124 (11.4%)	12 (1.1%)	0	3 (0.3%)	404 (37%)

^a^Percentages may not total 100% owing to rounding

### Prevalence of *Plasmodium* sporozoite infections in the mosquitoes

Of 914 *An*. *funestus* s.l tested for *Plasmodium* spp. infection, 13 were found positive for *P*. *falciparum* (Table 4). The majority of the infections were in *An*. *funestus* s.s. (*n* = 11). In addition, there were two *An*. *parensis* mosquitoes infected with *P*. *falciparum* (*n* = 2). No other *Plasmodium* species were detected, nor were any sibling species of *An*. *funestus* (besides *An*. *funestus* s.s. and *An*. *parensis*) found to be infected with *Plasmodium* spp.

**Table 4:** *Plasmodium* spp. prevalence in *Anopheles funestus* sibling species collected from 14 districts across mainland Tanzania

Region	District	No. tested	No. positive	Sibling species	*Plasmodium* spp.	Prevalence (%)
Dodoma	Chamwino	34	0	–	–	0
Kigoma	Kibondo	41	0	–	–	0
Kigoma	Kakonko	32	1	*An*. *funestus* s.s	*P*. *falciparum*	3.1
Tanga	Muheza	40	1	*An*. *funestus* s.s	*P*. *falciparum*	2.5
Kagera	Misenyi	48	3	*An*. *funestus* s.s	*P*. *falciparum*	6.3
Ruvuma	Tunduru	48	2	*An*. *funestus* s.s	*P*. *falciparum*	4.2
Morogoro	Ulanga (Kilisa)	21	0	–	–	0
Morogoro	Ulanga (Igumbiro)	84	3	*An*. *funestus* s.s	*P*. *falciparum*	3.6
Morogoro	Kilombero (Sululu)	70	0	–	–	0
Pwani	Bagamoyo	76	1	*An*. *funestus* s.s	*P*. *falciparum*	1.3
Mwanza	Misungwi	84	2	*An*. *parensis*	*P*. *falciparum*	2.4
Katavi	Tanganyika	84	0	–	–	0
Rukwa	Nkasi	84	0	–	–	0
Lindi	Mtama	84	0	–	–	0
Mtwara	Mahurunga	84	0	–	–	0
Total_a_		914	13 (1.4%)	N/A	N/A	N/A

^a^Mosquitoes collected from larval collections were not included in the *Plasmodium* spp. detection assay

### Transmission intensities mediated by *An*. *funestus* group

The annualized EIR estimates varied significantly across the regions. The highest EIR estimates were recorded in Kagera [108.3 infectious bites/person/year (ib/p/y)], Ruvuma (72.2 ib/p/y) and Morogoro regions (65.6 ib/p/y). Since no infected mosquitoes were collected in Dodoma, Rukwa, Lindi, Katavi and Mtwara, it was not possible to estimate EIRs from the *An*. *funestus* s.l collected in these regions (Table 5).

**Table 5:** Annual entomological inoculation rates owing to *Anopheles funestus* group by regions

Region	Trap nights	No. caught	Corrected biting rate	No. tested	No. positive	Sporozoite prevalence	Annual EIR (by *An*. *funestus*)
Dodoma	15	34	3.34	34	0	0	Not estimable^a^
Kigoma	30	73	3.57	73	1	0.014	18.2
Tanga	15	40	3.93	40	1	0.025	35.9
Kagera	15	48	4.71	48	3	0.063	108.3
Ruvuma	15	48	4.71	48	2	0.042	72.2
Morogoro	30	193	9.46	154	3	0.019	65.6
Pwani	15	76	7.45	76	1	0.013	35.4
Katavi	35	400	16.81	84	0	0	Not estimable
Rukwa	30	200	9.8	84	0	0	Not estimable
Lindi	30	989	48.49	84	0	0	Not estimable
Mtwara	44	1403	46.9	84	0	0	Not estimable

^a^The sampling in this study, aimed primarily at species identification and genomic analysis, was insufficient to conclusively exclude *Plasmodium* infections in entire regions where they were not detected. Therefore, regions with zero entomological inoculation rate (EIR) estimates are labelled as ‘Not estimable’, anticipating that future surveys may reveal non-zero prevalence rates

## Discussion

*Anopheles funestus* mosquitoes are among the most widespread, and yet also among the least studied species of malaria vectors. However, in recent years, there has been an increasing awareness that populations of *An*. *funestus* s.s., known for their high degree of anthropophily and now marked by significant pesticide resistance [35, 46], are becoming predominant in many malaria transmission areas, particularly in East and Southern Africa [59, 60]. In areas, such as south-eastern Tanzania, this species now mediates 85–98% of new malaria infections, even in villages where it is outnumbered by other species, such as *An*. *arabienesis* [31, 46, 61]. Today, the species composition and distribution of the *An*. *funestus* group, particularly in Tanzania, are well described. However, despite field collections regularly capturing several other members of the complex in many locations, the ecology and vectorial importance of these potential secondary vectors are poorly understood. This current study was therefore aimed at expanding on the existing knowledge towards understanding the vectorial role of *An*. *funestus* species across Tanzania mainland.

We found four known and previously reported sibling species of the *An*. *funestus* group (i.e. *An*. *funestus* s.s., *An*. *parensis*, *An*. *rivulorum* and *An*. *leesoni*), with *An*. *funestus* s.s. dominating malaria transmission across all the 12 regions surveyed. Moreover, 37% of the collected mosquitoes were not amplified by the available species-specific PCR assay [56] designed for the *An*. *funestus* group, despite being morphologically identified as *An*. *funestus* s.l. While this is a significantly high failure rate of the recommended PCR assays, similar non-amplification problems have been reported in previous studies, albeit at lower rates, including in south-eastern Tanzania [35, 45, 46]. Nonetheless, upon cloning and sequencing, it was confirmed that the ITS2 sequences were similar to that of *An*. *funestus* s.s. with the exception of polymorphisms present within the priming site of the common and widely used species-specific reverse primer. This PCR mis-priming is hereby considered the main reason for the high rates of non-amplification observed in this study; and may also have affected the aforementioned past studies. It is noteworthy, that all technologies based on PCR amplification, including higher throughput species identification multilocus amplicon panel approaches [62], will at times face similar issues because of the highly polymorphic genomes of Anopheline vector species. This suggests the need to continue improving the methods for identifying members of such species groups and complexes.

The incrimination of *An*. *parensis* with transmission of *P*. *falciparum* in this study provides only the third such report in Tanzania in the past 15 years [39, 63]. The two previous reports [39, 63] utilized CDC light traps, pyrethrum spray catch and aspirators for indoor sampling of host-seeking *An*. *parensis* mosquitoes, with nested PCR and enzyme linked immunosorbent assay (ELISA) as methods of *Plasmodium* sporozoite detection. Furthermore, the first report which was based on four specimens reported a 25% sporozoite rate [39], whilst the one conducted within the similar geographical area surrounding Lake Victoria as our present study and based on hundreds of samples reported 1.1% rate [63]. Collectively, these repeated observations suggest that *An*. *parensis* may be playing a modest but considerable role as a secondary malaria vector in Tanzania and should be further investigated to optimize the control of malaria transmission.

In this study, the two *Plasmodium*-infected *An*. *parensis* mosquitoes were found in the village of Ngaya in the Misungwi district in north-western Tanzania where long-lasting insecticidal nets (LLINs) were already widely implemented [63]. A 2018 study [64] focusing on *An*. *funestus* group species composition in several villages of the same district reported over 90% *An*. *funestus* s.s. and 6.5% *An*. *parensis*. While the aquatic ecology of *An*. *parensis* was not within the scope of this paper, studies in rural south-eastern Tanzania noted that *An*. *parensis* generally shared aquatic habitats with *An*. *funestus* s.s. and *An*. *rivulorum* (Kahamba et al. Unpublished data). Further studies are required to understand how vector control interventions might have been associated with the apparently higher importance of *An*. *parensis* in this location.

On the basis of our present findings there is a possibility that *An*. *parensis* may be contributing to residual malaria transmission, particularly in localities where *An*. *funestus* s.s. and other major vector species have been significantly impacted by chemical control interventions. This has previously been observed in the north-eastern part of South Africa, where *An*. *parensis* was reported to minimally contribute to residual malaria transmission, following an almost complete suppression of *An*. *funestus* s.s. following large-scale IRS implementation [38].

In previous studies, various sibling species within the *Anopheles funestus* group have been implicated as malaria vectors [37–39, 41, 43], resulting in multiple questions regarding the factors influencing their prevalence and roles in disease transmission. For instance, a study conducted in central Kenya reported significant densities of *An*. *parensis* inside human dwellings, though with a low human blood index [40]. In our present study, we also collected a significant number of resting *An*. *parensis* inside houses in the northern region. Additionally, our current findings, coupled with a previous study [65] conducted in the Muheza district of north-eastern Tanzania, which reported that over 60% of *An*. *parensis* caught inside houses had fed on humans despite the availability of cattle, signify the potential role of *An*. *parensis* as a contributor to the residual malaria transmission. Consequently, it will be necessary to extend our control efforts beyond the current indoor vector control interventions, to address not just *An*. *parensis*, but also other important species, such as *An*. *arabiensis*, which is also widespread in Tanzania [34] and tends to bite outdoors [66]. Additionally, there is a need for a thorough understanding of the ecology of *An*. *parensis* and other sibling species within the *An*. *funestus* group; as well as their responsiveness to current vector control interventions.

Annual entomological inoculation rates (EIR) were computed for different regions and were found to be the highest in areas where *An*. *funestus* s.s. dominate as the member of the *An*. *funestus* group, such as north-western and southern regions of Kagera and Ruvuma. Notably, Kigoma exhibited the lowest measurable EIR at 18.2 infectious bites per person per year (ib/p/yr) among all regions where infected mosquitoes were found. Among infected mosquitoes, *Plasmodium falciparum* was the only malarial parasite detected. However, it is essential to note that other *Plasmodium* species, such as *P*. *ovale* and *P*. *malariae*, have been previously reported in other country-wide surveys [67–69]. One limitation of this study was that the mosquito sampling, primarily designed for species identification and genomic analysis, was insufficient to definitively rule out *Plasmodium* infections in regions where none of the tested mosquitoes were found to be infected. Consequently, areas reporting zero EIR estimates are simply categorized as having non-estimable EIRs, rather than being considered as having no risk of malaria transmission. It is expected that expanded surveys would reveal non-zero prevalence rates within either the *An*. *funestus* group or the *An*. *gambiae* complex. Additionally, another limitation of the present study was the inability of the available species-specific PCR assay [56] designed for the *An*. *funestus* group, to identify 37% of the collected mosquitoes that had otherwise been morphologically identified as *An*. *funestus*.

## Conclusions

This study underscores the pivotal role of the *An*. *funestus* group in malaria transmission with a particular focus on the prominent *An*. *funestus* s.s. Additionally, the study sheds light on the lesser-studied sibling species, *An*. *parensis*, which is identified here, for the third time, as playing a role in the transmission of *Plasmodium falciparum*. Challenges in PCR amplification owing to ITS2 region polymorphisms highlight the limitations of current molecular tools for distinguishing species within the Funestus group. This study contributes to the body of knowledge on malaria vector composition and distribution in Tanzania and emphasizes the critical need for the adaptation of vector control interventions to regional specificities in malaria transmission dynamics. More importantly, the findings call for a deeper investigation into the ecology and vectorial capacity of secondary vectors to enhance malaria control strategies.

### Supplementary Information

Additional file 1: (A) An example sequence from NCBI (approximately 844 base pairs including primers) with accession number JN994135.1, comprising of partial sequences of 5.8S and 28S ribosomal RNA genes flanking the internal transcribed spacer 2 region. The outer forward and reverse primer sequences for the complete ITS2 region with 5.8S and 28S rRNA genes flanks are highlighted (green and yellow highlights). The reverse primer specific to *An*. *funestus* s.s. in the species identification assay is also shown (dark green). (B), (C) and (D) represent the same region cloned and sequenced from non-amplified samples which revealed polymorphisms within different sections of the reverse primer’s priming region (red).

## Data Availability

All data supporting the conclusions of this article are provided within the text, including the GenBank accession numbers PP853609, PP853610 and PP853611.

## Abbreviations

ITN: Insecticide treated bed net
IRS: Indoor residual spraying
PCR: Polymerase chain reaction
CDC: Centres for Disease Control and Prevention
EDTA: Ethylene diamine tetra acetic acid
ITS: Internal transcribed spacer
HLC: Human landing catch
EIR: Entomological inoculation rate
BLASTn: Basic local alignment search tool for nucleotides
NCBI nt: National centre for biotechnology information nucleotide
