# Supplementary material for: Entomological survey of sibling species in the Anopheles funestus group in Tanzania confirms the role of Anopheles parensis as a secondary malaria vector
Source: Parasit Vectors. 2024 Jun 17;17:261. doi: 10.1186/s13071-024-06348-9 (PMC11181546; doi:10.1186/s13071-024-06348-9)
Supplement: Supplementary file 1 — Additional file 1: (A) An example sequence from NCBI (approximately 844 base pairs including primers) with accession number JN994135.1, comprising of partial sequences of 5.8S and 28S ribosomal RNA genes flanking the internal transcribed spacer 2 region. The outer forward and reverse primer sequences for the complete ITS2 region with 5.8S and 28S rRNA genes flanks are highlighted (green and yellow highlights). The reverse primer specific to An. funestus s.s. in the species identification assay is also shown (dark green). (B), (C) and (D) represent the same region cloned and sequenced from non-amplified samples which revealed polymorphisms within different sections of the reverse primer’s priming region (red). [file 13071_2024_6348_MOESM1_ESM.docx]

**Additional file 1:** (A) An example sequence from NCBI (approximately 844 base pairs including primers) with accession number JN994135.1, comprising of partial sequences of 5.8S and 28S ribosomal RNA genes flanking the internal transcribed spacer 2 region. The outer forward and reverse primer sequences for the complete ITS2 region with 5.8S and 28S rRNA genes flanks are highlighted (green and yellow highlights). The reverse primer specific to *An. funestus* s.s. in the species identification assay is also shown (dark green). (B), (C) and (D) represent the same region cloned and sequenced from non-amplified samples which revealed polymorphisms within different sections of the reverse primer’s priming region (red).
